# A Viral Polymerase Inhibitor Reduces Zika Virus Replication in the Reproductive Organs of Male Mice

**DOI:** 10.3390/ijms20092122

**Published:** 2019-04-29

**Authors:** Sofie Jacobs, Leen Delang, Eric Verbeken, Johan Neyts, Suzanne J.F. Kaptein

**Affiliations:** 1 KU Leuven Department of Microbiology and Immunology, Rega Institute for Medical Research, Laboratory of Virology and Chemotherapy, 3000 Leuven, Belgium; sofie-jacobs@kuleuven.be (S.J.); leen.delang@kuleuven.be (L.D.); suzanne.kaptein@kuleuven.be (S.J.F.K.); 2 Laboratory of Pathology, 3000 Leuven, Belgium; eric.verbeken@uzleuven.be

**Keywords:** antivirals, sexual transmission, Zika virus

## Abstract

In humans, Zika virus and viral RNA have been detected in semen up to 2.2 months and 6 months post infection (pi), respectively. Although the contribution of sexual transmission to the spread of ZIKV is too low to sustain an outbreak, it can increase the risk of infection and the epidemic size as well as prolong the duration of an outbreak. In this study, we explored the potential of antivirals to serve as an effective strategy to prevent sexual transmission. Male AG129 mice infected with a ZIKV isolate from Suriname were treated with the nucleoside analog, 7-deaza-2′-*C*-methyladenosine (7DMA), that was previously shown to be efficacious in reducing ZIKV viremia and delaying ZIKV-induced disease in mice. Following treatment, viral RNA and infectious virus titers were consistently reduced in the male reproductive organs compared to vehicle-treated mice. This reduction of ZIKV loads in the testis was confirmed by the detection of lower levels of ZIKV antigens. Our data illustrate the value of this mouse model to validate the efficacy of new potential ZIKV drugs at the level of the male reproductive system.

## 1. Introduction

Zika virus (ZIKV) is a re-emerging, arthropod-borne virus (arbovirus), belonging to the family of *Flaviviridae*. After being isolated from a human for the first time in 1952, only sporadic cases of human ZIKV infections were reported and there were no reports on transmission outside Africa or Southeast Asia [1]. The first recorded ZIKV outbreak occurred in 2007 on Yap Island, Federated States of Micronesia, where 73% of the residents became infected [1]. No further transmission was identified in the Pacific until October 2013, when an outbreak occurred in French Polynesia, followed by the emergence of ZIKV on other Pacific islands [2]. In May 2015, ZIKV autochthonous cases were identified for the first time in the Americas [3]. By January 2018, the number of cumulative Zika cases in the Americas exceeded 800,000 [4], demonstrating that flaviviruses can cause explosive and large outbreaks.

Prior to the outbreak in French Polynesia, ZIKV infections were mainly reported to be asymptomatic or to result in a febrile self-limiting disease, characterized by mild fever, headache, rash, arthralgia, myalgia, and conjunctivitis [5]. However, during the French Polynesia outbreak, an unusual increase in Guillain–Barré syndrome (GBS, an autoimmune disease causing acute or subacute flaccid paralysis) was reported that coincided both temporally and spatially with a peak in the incidence of ZIKV infections [6,7]. Moreover, during the ZIKV outbreak in the Americas, an increased incidence of microcephaly among newborns was observed [8]. Subsequent retrospective studies revealed a similar, unusual increase in microcephaly cases during the 2013–2014 outbreak in French Polynesia [9]. By 2016, the accumulating evidence supporting a link between ZIKV infection in pregnant women and congenital neurological abnormalities led the WHO to declare ZIKV a Public Health Emergency of International Concern [10,11,12].

ZIKV is predominantly transmitted to humans through the bite of *Aedes* mosquitoes, primarily *Ae. aegypti* and secondarily *Ae. albopictus* [13]. However, recent studies showed that ZIKV can also be sexually transmitted between humans, with male to female transmission being the most common. Infectious virus has been detected in semen for up to 69 days post infection (pi) [14]. Mathematical models predict that the contribution of sexual transmission to the spread of ZIKV is 3–4.8% [15,16]. The low contribution (~1%) of sexually transmitted ZIKV cases to the overall epidemiology was confirmed in recent reviews [17,18]. These models also suggest that, although the contribution of sexual transmission is too low to sustain an outbreak, it can increase the risk of infection and epidemic size as well as prolong the duration of an outbreak. Therefore, prevention and control measures should not only focus on mosquito-borne transmission, but also on the sexual transmission route [14,16]. To avoid sexual transmission, both symptomatic and asymptomatic male patients and travelers returning from areas with a high risk of ZIKV infection are recommended to practice safe sex for at least six months [19]. Furthermore, infected female partners or women returning from an endemic area should wait at least eight weeks before considering pregnancy [19].

Due to the unavailability of antivirals and vaccines against ZIKV infections, patients are currently being treated symptomatically and mosquito-borne transmission is prevented by applying individual personal protective measures and vector control strategies. We previously reported on the establishment of a robust AG129 mouse model of ZIKV infection with involvement of the male reproductive tract that was validated to evaluate the efficacy of candidate antivirals in inhibiting ZIKV replication [20]. Here, we describe the utility of this model to evaluate the use of antiviral molecules as a strategy against sexual transmission of ZIKV by reducing the viral load in male reproductive organs.

## 2. Results

**Figure 1:**
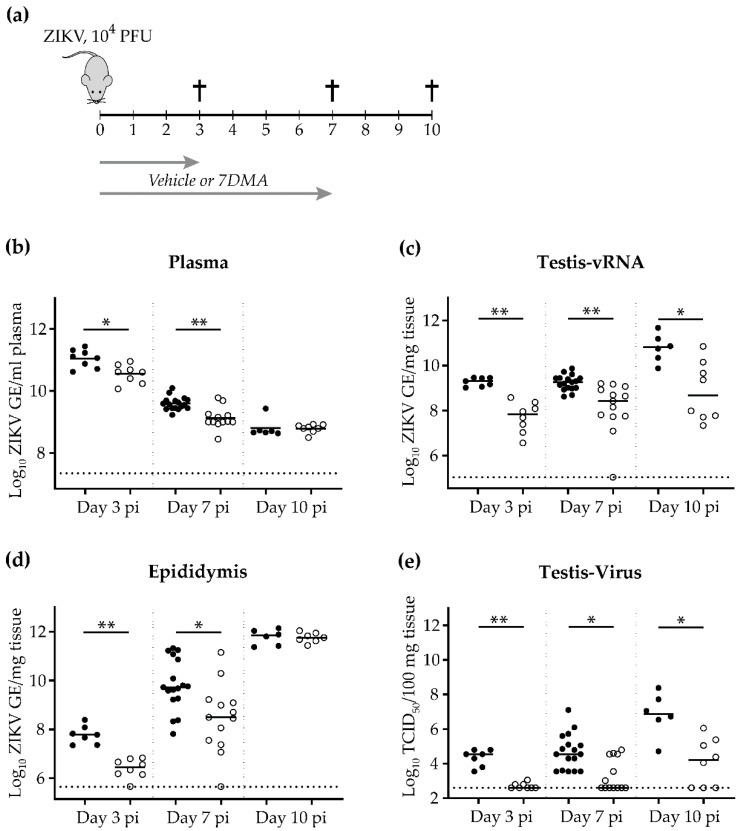
7DMA treatment reduces ZIKV replication in male reproductive organs. Male AG129 mice (6-12 weeks of age) were inoculated intraperitoneally with 10^4^ PFU of ZIKV (SL1602, Suriname isolate). (**a**) Schematic representation of the study design. Starting from the day of infection (i.e., day 0), mice were treated once daily with 50 mg/kg/dose of 7DMA or vehicle via oral gavage for 7 consecutive days. The inhibitory effect of 7DMA on ZIKV replication in plasma (**b**), the testis (**c,e**), and the epididymis (**d**) is compared between vehicle-treated mice (black, *n* = 8, 17, and 6) and mice treated with 7DMA (white, *n* = 8, 13, and 8) at day 3, 7, and 10 pi, respectively. Data are presented as medians and statistical analysis was performed using the Mann-Whitney U test, * = *p* ≤ 0.008, ** = *p* ≤ 0.0006 (Graphpad software). GE; genome equivalents. The dotted line represents the limit of detection. Data from day 7 pi are from two independently performed experiments.

We previously demonstrated the ability of 7-deaza-2′-*C*-methyladenosine (7DMA) to delay ZIKV-induced disease in AG129 mice when administered at the time of or two days prior to infection [20]. Here, we evaluated the antiviral efficacy of 7DMA on ZIKV loads in the male reproductive organs (i.e., testis and epididymis). Male AG129 mice (6-12 weeks of age) were infected intraperitoneally with 10^4^ PFU of ZIKV SL1602 (Suriname isolate). Starting from the day of infection (i.e., day 0), mice were treated once daily with 50 mg/kg/dose of 7DMA or vehicle via oral gavage for 7 consecutive days (Figure 1a). At day 3, 7, and 10 pi, mice were sacrificed and viral RNA was extracted from plasma, testis, and epididymis. Infectious virus titers from the testis were determined by means of end-point titrations. The viremia in vehicle-treated mice significantly decreased from day 3 to day 10 pi (Figure 1b), which is in accordance with the normal progression of a ZIKV infection in mice [20,21]. The viral RNA load in the reproductive organs of vehicle-treated mice increased from day 3 to day 10 pi (Figure 1c–e), consistent with previous reports [22]. Compared to vehicle-treated mice, the viral RNA load in the plasma and reproductive organs of 7DMA-treated mice was consistently reduced at day 3 and day 7 pi (Figure 1b–e). At day 7 pi, almost 70% of the 7DMA-treated mice had undetectable levels of infectious virus in their testis compared to none of the vehicle-treated mice (Figure 1e), indicating that a single daily dose of 7DMA is efficacious in inhibiting ZIKV replication in the testis of male mice.

**Figure 2:**
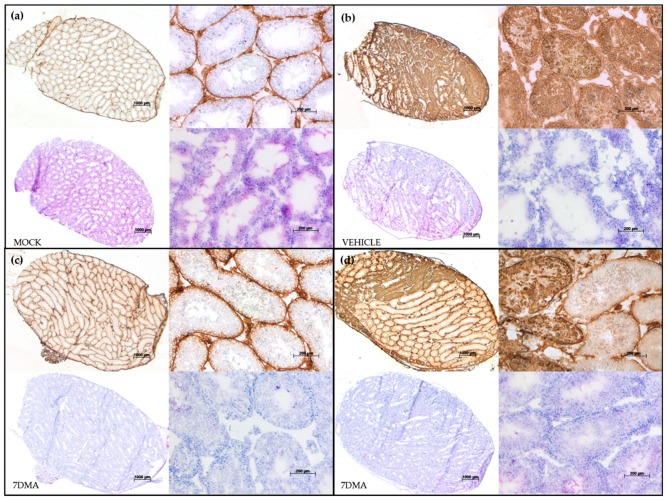
Testicular levels of ZIKV antigens are reduced after 7DMA treatment as visualized by histopathological staining. Inoculation and treatment of AG129 mice was performed as described in Figure 3. The presence of ZIKV antigens (top panels in each quadrant) and inflammation (bottom panels in each quadrant) in the testis at day 10 pi is compared between mock-infected mice (**a**) and ZIKV-infected mice treated with vehicle (**b**) or 7DMA (**c**,**d**). The top two panels in each quadrant show antibody staining for the ZIKV envelope protein. The bottom two panels in each quadrant show hematoxylin and eosin staining. Panels on the left in each quadrant show a complete cross section of the testis. Panels on the right of each quadrant show a close up of the complete cross section of the same quadrant. The scale bars are 1000 µm and 200 µm for cross sections and close ups, respectively.

At day 10 pi, levels of viral RNA in plasma and epididymal tissue in 7DMA-treated mice did not differ significantly from those in vehicle-treated mice (Figure 1b,d). In contrast, levels of viral RNA and infectious virus in testicular tissue were significantly lower in 7DMA-treated mice than in vehicle-treated mice (Figure 1c,e). This is corroborated by the reduced expression of ZIKV antigens in the testis of 7DMA-treated mice at day 10 pi (Figure 2c,d; top panels in each quadrant) compared to the testis of vehicle-treated mice, which abundantly expressed ZIKV antigens (Figure 2b). However, 7DMA was not able to completely block the expression of ZIKV antigens in the testis of all treated mice, as shown in Figure 2d (compared to Figure 2c), demonstrating the relative weak potency of 7DMA. Irrespective of the treatment regimen, signs of increased inflammation (i.e., inflammatory cell infiltration) as a result of ZIKV infection were absent at day 10 pi in the testis of all ZIKV-infected mice, as is evident from the hematoxylin and eosin (H&E) stained sections (Figure 2, bottom panels in each quadrant). Together, these findings demonstrate that an antiviral, such as 7DMA, is able to maintain a reduced testicular viral load beyond the end of treatment. However, the antiviral potency of 7DMA is not sufficient to also maintain reduced viral levels in the epididymis (and presumably semen) at a later time point pi. Future antiviral drug candidates should be sufficiently potent in inhibiting viral replication in the testis and epididymis of infected mice, both early and late during a ZIKV infection.

**Figure 3:**
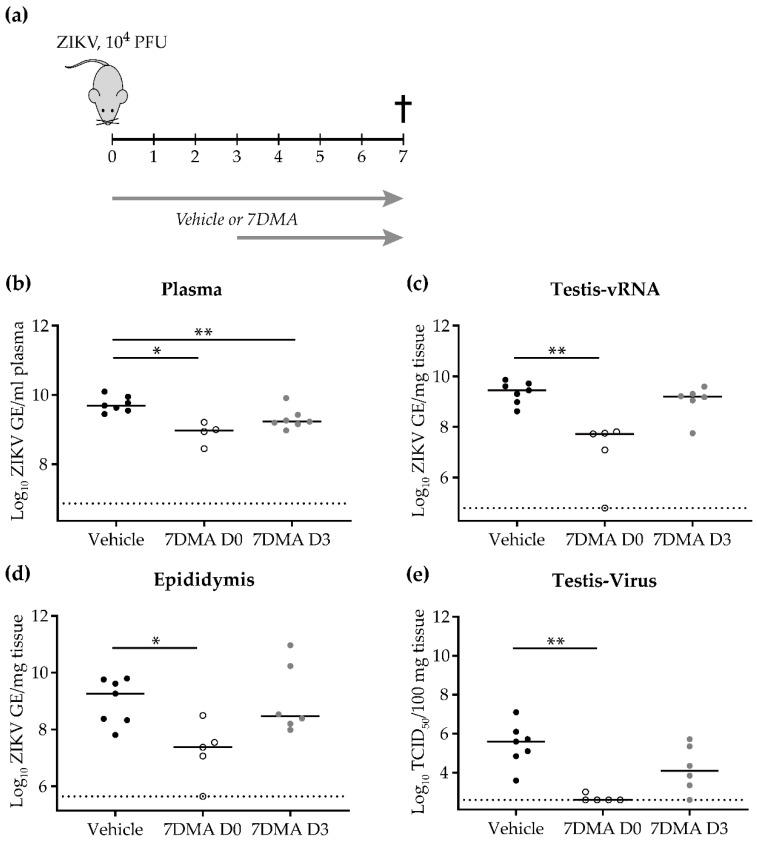
Early 7DMA treatment is required to efficiently inhibit ZIKV replication in male reproductive organs. (**a**) Schematic representation of the study design. Male AG129 mice (6-10 weeks of age) were inoculated intraperitoneally with 10^4^ PFU of ZIKV. The inhibitory effect of 7DMA on ZIKV replication in plasma (**b**), the testis (**c**,**e**), and the epididymis (**d**) at day 7 pi is compared between vehicle-treated mice (black, *n* = 7) and mice treated with 7DMA starting either at the day of infection (white, *n* = 5) or at day 3 pi (grey, *n* = 7). Data are presented as medians and statistical analysis was performed using the Mann-Whitney U test, * = *p* < 0.05, ** = *p* ≤ 0.006 (Graphpad software). GE; genome equivalents. The dotted line represents the limit of detection.

Next, we evaluated the antiviral efficacy of 7DMA when treatment was initiated at a later time point pi. To this end, male AG129 mice (6-10 weeks of age) were inoculated intraperitonially with 10^4^ PFU of ZIKV SL1602. Animals received the first dose of 7DMA (*n* = 5 and 8) or vehicle (*n* = 7) either one hour prior to infection or at day 3 pi (Figure 3a). Mice were treated once daily with 50 mg/kg/dose of 7DMA or vehicle via oral gavage until day 7 pi. At day 7 pi, viral RNA levels in plasma and reproductive organs were significantly reduced in mice that received early 7DMA treatment compared to vehicle-treated mice (Figure 3b–e). Similarly, infectious virus titers in the testis were significantly lower compared to those in vehicle-treated mice (Figure 3e). In contrast, levels of ZIKV RNA and infectious virus in mice from the delayed treatment group did not differ significantly from those in vehicle-treated mice (Figure 3b–e). These results indicate that early treatment with 7DMA is required for effective inhibition of ZIKV replication in the male reproductive system.

## 3. Discussion

ZIKV generally does not replicate nor cause disease in wild-type mice, hence models to study ZIKV pathogenesis of the male reproductive tract and sexual transmission typically involve immunodeficient mice, such as A129, *Ifnar1^−/−^* (both lacking IFN-α/β receptors), and AG129 mice (lacking IFN-α/β and IFN-γ receptors) [14]. Infection of male AG129 mice with the ZIKV isolate SL1602 from Suriname resulted in high viral RNA levels in testicular and epididymal tissues at day 3 pi [23]. Viral RNA levels in the testis and epididymis increased even further until the end of the study at day 10 pi. Similarly, increasing levels of infectious ZIKV in the testis from day 3 (4.5log_10_ TCID50/100 mg tissue; median) until day 10 pi (6.9log_10_ TCID50/100 mg tissue; median) were observed. This is in line with previous studies, in which increasingly high levels of viral RNA and infectious virus were observed in the testis of immunocompromised mice (*Ifnar^−/−^* or A129 mice) using contemporary ZIKV isolates (strain H/PF/2013 from French-Polynesia or strain FSS13025 from Cambodia) [22,24]. In contrast to previous studies, we did not observe any signs of testicular atrophy. We observed the mice for 10 days, which may have been too short a period to detect tissue damage since others reported distinct testicular damage much later after infection, i.e., day 21 and day 15-30 pi [25,26,27]. Other reasons may be differences in the mouse species used (AG129 mice versus *Ifnar^−/−^* or wild-type mice that were treated with an interferon antibody) and/or the ZIKV strain [25,26,27].

We did not engage in identifying the target cells of ZIKV replication in the testis/reproductive male tract since studies on this topic have already been published [25,26,27]. A study on the persistence of ZIKV in seminal fluids of male AG129 mice revealed the presence of infectious ZIKV in the ejaculates from day 7 to day 21 pi [28]. The highest viral titer per ejaculate (5.6log_10_ PFU) was observed at day 8 pi. During the two week window of infectivity, sexual transmission from ZIKV-infected male mice to uninfected females was observed, with transmission occurring in 50% of all mating events [28]. Another study demonstrated the permissiveness of male murine germ cells of both wild-type and *Ifnar1^−/−^* mice to infection with various ZIKV isolates [29]. The hypothesis that germ cells are potential target cells of ZIKV replication was confirmed using an ex vivo model of primary human testicular tissue [29]. However, reports on sexual transmission via vasectomized men suggest that the virus does not only reside in germ cells [30,31]. Indeed, active ZIKV replication was observed in human prostate cells [32], indicating that the prostate and seminal vesicles may serve as potential ZIKV reservoirs, which can facilitate sexual transmission.

An alternative way to prevent or lower the risk of ZIKV sexual transmission is through antiviral treatment. We thus focused on establishing an in vivo model to evaluate the efficacy of antiviral molecules to lower or even fully inhibit ZIKV replication in the reproductive tract of male mice. We previously reported on the ability of the viral polymerase inhibitor, 7DMA, to delay virus-induced disease in AG129 mice infected with the prototype ZIKV MR766 strain [20]. In the present study, the inhibitory potential of 7DMA was tested against a ZIKV strain from Suriname at the level of the male reproductive tract. Significantly lower levels of viral RNA and infectious virus were observed in the testis of mice that were treated with 7DMA starting at the day of infection compared to vehicle-treated mice, although some mice were less responsive to the antiviral treatment. This is likely due to interindividual differences in the susceptibility to ZIKV infection and/or differential pharmacological responses to 7DMA. Interestingly, reduced levels of virus were observed in the testis, but not in the epididymis, of 7DMA-treated mice until three days after termination of antiviral treatment. These observations were corroborated by lower expression levels of ZIKV antigens in the testicular tissue. Delayed start of treatment did not have a significant inhibitory effect on the levels of viral RNA and infectious virus. Although 7DMA was unable to completely suppress ZIKV replication in male reproductive tissues, due to the relatively weak potency of the compound [20], the data presented here validate our model as a tool to examine the antiviral competence of new candidate drugs targeted at preventing ZIKV sexual transmission. It also demonstrates that antiviral treatment can help to maintain a lower viral load in the male reproductive tract, which could aid in the prevention of sexual transmission.

## 4. Materials and Methods 

### 4.1. Cells, Virus, and Compounds

C6/36 mosquito cell cultures (ATCC CRL-1660) were maintained in Leibovitz’s L-15 medium supplemented with 10% fetal bovine serum (FBS), 1% (1×) non-essential amino acids (NEAA), 10 mM HEPES buffer, 100 units/mL penicillin, and 100 µg/mL streptomycin (Penicillin-Steptomycin) at 28 °C without CO_2_. Vero E6 cell cultures (Vero C1008; ATCC CRL-1586) were maintained in MEM Rega-3 medium supplemented with 10% FBS, 2 mM L-glutamine, and 0.075% sodium bicarbonate. For cell culture assays that involved virus or virus infected material, the concentration of FBS in the medium was reduced to 2% (2% medium). All tissue culture media and supplements were obtained from Gibco, Thermo Fisher Scientific (Merelbeke, Belgium).

ZIKV (SL1602, Suriname isolate) was obtained from Prof. Martijn van Hemert, Leiden University Medical Center, Leiden, The Netherlands. Lyophilized virus was reconstituted in 2% MEM Rega-3 medium and virus stocks were generated on C6/36 mosquito cell cultures as described before [20]. The aforementioned cell types tested negative for mycoplasma. 

7-deaza-2′-*C*-methyl-d-adenosine (7DMA) was purchased from Carbosynth (Berkshire, UK).

### 4.2. In Vivo Evaluation of 7DMA Against ZIKV Replication in AG129 Mice

All experiments were performed with the approval and under the guidelines of the Ethical Committee of the University of Leuven (P087-2014). Male AG129 mice (deficient in both interferon (IFN)-α/β and IFN-γ receptors) of 6–12 weeks of age were treated once daily with either 50 mg/kg/day of 7DMA resuspended in 0.4% sodium carboxymethylcellulose (CMC-Na) or vehicle (0.4% CMC-Na) via oral gavage. One hour following the first treatment, all mice were infected intraperitoneally (ip) with 10^4^ PFU of ZIKV SL1602 in 200 µL. Mice were treated with the 7DMA or vehicle for 7 consecutive days. Mice were observed daily for body weight change and the development of virus-induced disease. In the case of >20% body weight loss and/or severe illness, mice were euthanized using Doléthal (Vétoquinol, Aartselaar, Belgium). Mice were sacrificed at day 7 or day 10 pi. Blood was collected by cardiac puncture and tissues (testis and epididymis) were collected after transcardial perfusion using PBS and immediately placed on dry ice. Tissues were stored at −80 °C until further evaluation.

### 4.3. Tissue RNA Isolation and Quantitative Reverse Polymerase Chain Reaction (qRT-PCR)

Sections of whole tissue were transferred to 2 mL Precellys tubes containing 2.8 mm zirconium oxide beads (Bertin Instruments, Montigny-le-Bretonneux, France). Subsequently, RLT lysis buffer (RNeasy Mini Kit, Qiagen, Antwerp, Belgium) was added at a ratio of 100 µL of buffer per 5 mg of tissue. Tissue homogenates were prepared using an automated homogenizer (Precellys24, Bertin Instruments). Homogenates were cleared by centrifugation and total RNA was extracted from the supernatant using the RNeasy Mini Kit (Qiagen), according to the manufacturer’s protocol. The NucleoSpin RNA kit (Macherey-Nagel, Eupen, Belgium) was used to isolate viral RNA from serum samples. For both kits, RNA was eluted in 50 µL of RNase-free water. During qRT-PCR, the ZIKV E protein encoding region (nucleotides 1193–1269) was amplified using the primers, 5′-CCGCTGCCCAACACAAG-3′ (forward) and 5′-CCACTAACGTTCTTTTGCAGAC AT-3′ (reverse), and a Double-Quenched Probe 5’-6-*FAM*/AGCCTACCT/*ZEN*/TGACAAGCAATCA GACACTCAA/3′*IABkFQ* (Integrated DNA Technologies, IDT, Leuven, Belgium). Viral copy numbers were quantified using serial dilutions of a viral RNA template that was isolated from the ZIKV stock that was also used to inoculate the mice.

### 4.4. Virus End-Point Titration by Cell Culture Infectious Dose 50% (TCID50) Assay

To determine infectious viral titers from tissue, tissue homogenates were prepared in 2% medium. To this end, sections of whole tissue were transferred to 2 mL Precellys tubes containing 2.8 mm zirconium oxide beads and 2% medium was added at a ratio of 100 µL of medium per 5 mg of tissue. Tissue homogenates were prepared using an automated homogenizer and were cleared by centrifugation. The infectious viral titers in tissues were subsequently determined by a TCID50 assay on Vero E6 cells. To this end, Vero E6 cells were seeded at a density of 10^4^ cells/well in a 96-well plate in 100 µL of 2% medium and allowed to adhere overnight. The following day, 2% medium was added to all wells to a total volume of 180 µL. In total, 20 µL of homogenate supernatant was added to the first well to a final dilution of 1/10. Then, 20 µL was systematically transferred to the subsequent well to obtain a 10-fold serial dilution. Following 7 days of incubation at 37 °C, CPE was determined by microscopic evaluation. TCID50 values were calculated using the Reed and Muench method [33]. The limit of detection is determined by the minimal amount of positive wells in the first dilution required to obtain a TCID50 value using the Reed and Muench method.

### 4.5. Histopathology

Testes were collected and placed on dry ice before storage at −80 °C. Frozen testes were embedded in KP cryocompound, cut into 5 µm sections, and fixed in acetone. Sections were stained with hematoxylin-eosin (H&E) to detect signs of inflammation and tissue damage. In addition, sections were stained with the anti-Flavivirus Group Antigen Antibody, clone D1-4G2-4-15 (Millipore, Overijse, Belgium), to detect the ZIKV-encoded envelope (E) protein. The primary antibody binding was visualized using the BOND polymer refine detection kit (Leica, Diegem, Belgium), according to the manufacturer’s protocol. In brief, primary antibody staining was followed by incubation with post-primary rabbit anti-mouse IgG antibodies and secondary goat anti-rabbit IgG antibodies conjugated with horseradish peroxidase (HRP). HRP staining was performed with 3,3′-diaminobenzidine tetrahydrochloride hydratediaminobenzidine (DAB, brown staining).
